# Secondary Chondrosarcoma Presenting with Symptoms Similar to Thoracic Outlet Syndrome

**DOI:** 10.1155/2018/9347145

**Published:** 2018-03-12

**Authors:** Hiroshi Kobayashi, Masachika Ikegami, Tetsuo Ushiku, Masaki Anraku, Takahiro Ohki, Yusuke Shinoda, Sakae Tanaka, Hirotaka Kawano

**Affiliations:** ^1^Department of Orthopaedic Surgery, Faculty of Medicine, The University of Tokyo, 7-3-1 Hongo, Bunkyo-ku, Tokyo 113-8655, Japan; ^2^Department of Pathology, Faculty of Medicine, The University of Tokyo, 7-3-1 Hongo, Bunkyo-ku, Tokyo 113-8655, Japan; ^3^Department of Thoracic Surgery, Faculty of Medicine, The University of Tokyo, 7-3-1 Hongo, Bunkyo-ku, Tokyo 113-8655, Japan; ^4^Department of Orthopaedic Surgery, Teikyo University School of Medicine, 2-11-1 Kaga, Itabashi-ku, Tokyo 173-8606, Japan

## Abstract

Thoracic outlet syndrome (TOS) is caused by heterogeneous factors that compress the brachial plexus and subclavian artery; tumor is rarely a cause of TOS. Here, we present the case of a 26-year-old man with secondary chondrosarcoma arising from osteochondroma of the left clavicle causing TOS, with a direct compression of the brachial plexus and subclavian artery. Immediately after surgery, the symptoms of TOS reduced. To our knowledge, this is the first case of a secondary chondrosarcoma of the clavicle causing TOS, which is possibly the key symptom for diagnosing malignant transformation of osteochondroma of the clavicle.

## 1. Introduction

Thoracic outlet syndrome (TOS) is caused by compression of the subclavian artery, vein, or brachial plexus. The compression may be caused by various pathophysiologies. In fact, a few cases reported a tumor of the first rib as the cause of TOS [1, 2].

Secondary chondrosarcoma occurs in 0.5%–1% of patients with a solitary osteochondroma. Malignant transformation of osteochondroma is more common in hereditary multiple exostosis (HME) [3]. Common symptoms caused by malignant transformation of osteochondroma are increasing size of the palpable mass, pain around the tumor, and limitation of the range of motion of the adjacent joint. To our knowledge, there has been only one report of a tumor occurring at the clavicle causing TOS, but none have reported TOS as the manifestation of malignant transformation of osteochondroma [4].

We report a case with a secondary chondrosarcoma of the clavicle causing TOS. Tumor resection resolved both the neurologic symptoms and restriction of the range of motion of the shoulder.

## 2. Case Presentation

A 26-year-old man was referred to our hospital with complaints of progressive restriction of the range of motion of his left shoulder and worsening pain and dysesthesia of the left arm and hand. Eight years prior to presentation, he was diagnosed with HME in our hospital, and an osteochondroma of the right femur was resected. He had never been treated as he had not experienced any discomfort since that.

**Figure 1:**
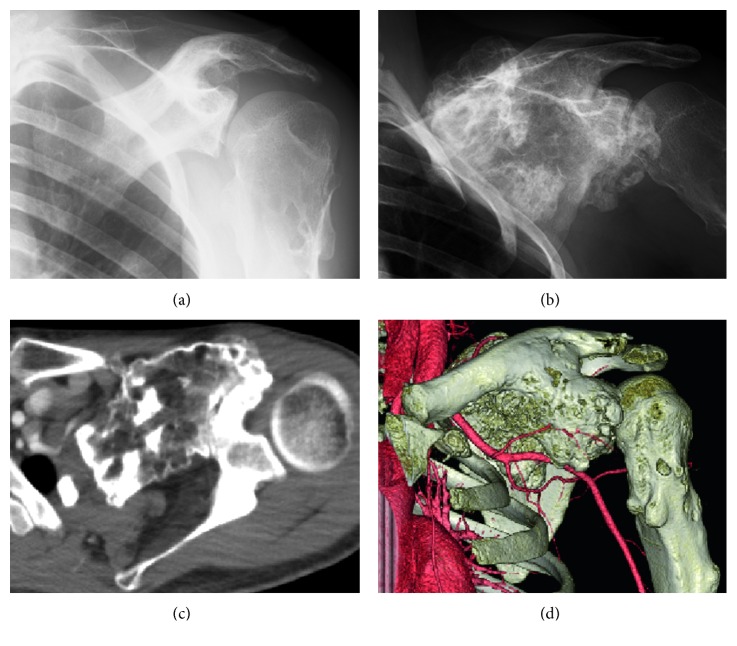
Anteroposterior radiographs of the left shoulder (a) 8 years before the presentation and (b) at presentation, showing that the tumor had apparently increased. (c) Axial computed tomography (CT) view of the left shoulder shows the tumor attached to the coracoid process. 3D reconstruction of the left shoulder illustrates structures in (d).

**Figure 2:**
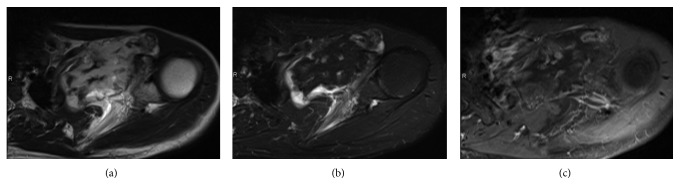
(a) T1-weighted magnetic resonance image (MRI). (b) T2-weighted MRI of fat suppression. (c) Contrast-enhanced T1-weighted preoperative MRI of fat suppression, axial view, showing a well-demarcated osseous lesion in the retroclavicular space with a thickened cartilaginous cap at the periphery of the tumor.

**Figure 3:**
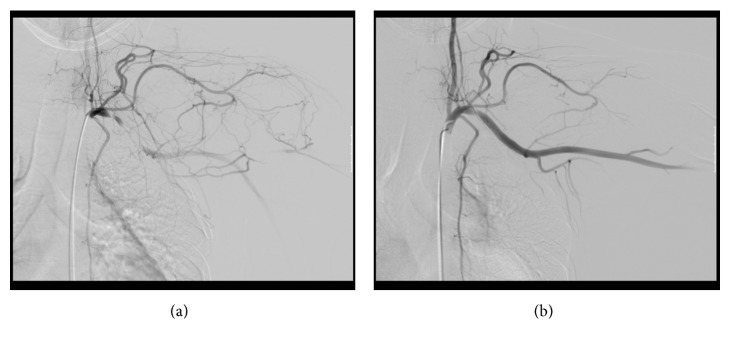
Angiography of the subclavian artery. (a) At the neutral position. (b) At 60° elevation of the left arm, the obstruction of the subclavian artery is released.

Physical examination revealed a 10 × 10 cm fixed, hard mass in the left infraclavicular lesion, and the range of motion of the left shoulder was limited (flexion and abduction was 90°). Dysesthesia and radiating pain to the left arm and hand were observed when the arm position was dropped down for a few minutes and were relieved by elevating the arm. Slight weakness of the extensor digitorum communis and extensor carpi radialis brevis muscles was detected. Adson's test for TOS was positive with loss of radial pulse and numbness in the left arm and hand, and Wright's hyperabduction test was negative. Plain radiographs of the left shoulder showed that the bony mass adjacent to the clavicle had markedly increased compared with 8 years previously (Figures 1(a) and 1(b)). Computed tomography of the left shoulder showed a bony lesion involving the left clavicle protruding in the posteroinferior direction, adhering to the coracoid process of the scapula; the subclavian artery was located under the tumor (Figures 1(c) and 1(d)). Magnetic resonance imaging (MRI) of the left shoulder revealed a maximal cartilaginous cap thickness of 1.7 cm, and T2-weighted and slightly gadolinium-enhanced MRI showed a high signal intensity of the cartilaginous cap (Figure 2). Combined with the increase in size after skeletal maturity, we diagnosed the tumor as a malignant transformation of osteochondroma. Angiography showed that elevating the left arm dissolved the disruption of the subclavian artery (Figure 3). These findings indicated that secondary chondrosarcoma had caused TOS by obstructing the subclavian artery, and the restricted range of motion in the left shoulder was caused by adhesion of the tumor to the scapula through the coracoid process.

**Figure 4:**
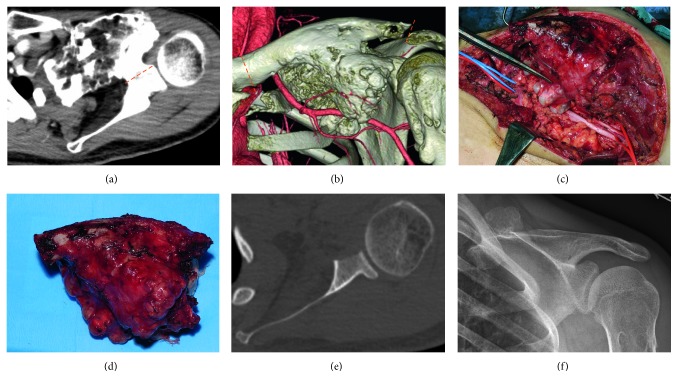
(a) Resection line of the coracoid process. (b) Resection line of the clavicle. (c) Intraoperative findings show that the subclavian artery and brachial plexus are compressed by the tumor. (d) Resected specimen. (e) Postoperative axial computed tomography view shows that the coracoid process is resected and the glenohumeral joint is preserved. (f) Anteroposterior view of the left shoulder 3 years after surgery shows that the tumor is resected from the clavicle and there is no recurrence.

The surgical procedure was performed through a transverse incision in line with the superior border of the clavicle, with osteotomy 3 cm from the proximal edge of the clavicle, and disarticulation of the acromioclavicular joint. The subclavian vessels and brachial plexus had been compressed downward by the tumor, and the compression worsened when his left arm was dropped as shown in the angiography. Because of this, we ensured that the arm remained abducted, and the subclavian vessels and brachial plexus were released easily because these structures were separated by the thin membrane enveloping the tumor when the tumor was resected en bloc. The clavicle was resected almost in entirety, and the coracoid process was resected from the scapula at its base (Figures 4(a)–4(d)).

**Figure 5:**
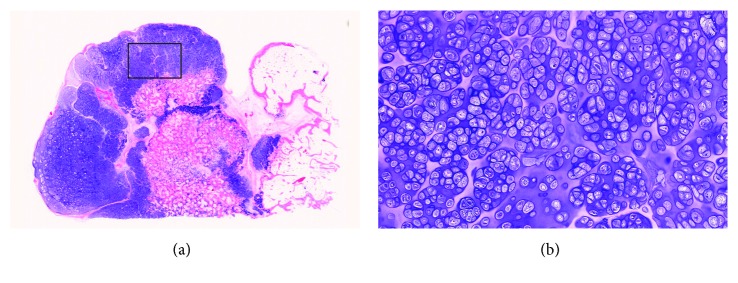
Histology of the resected tumor with hematoxylin and eosin stain. (a) On low-power view, the tumor has three layers: superficial perichondrium, thickened cartilage cap with lobular architecture, and endochondral ossification in the base. (b) On higher magnification (square area in (a)), increased cellularity and mild nuclear atypia of the neoplastic chondrocytes are noted.

On histological examination, the tumor was determined to be composed of cartilaginous tissue with mild nuclear atypia and increased cellularity showing a lobular growth pattern separated by fibrous bands (Figure 5). The histologic features were consistent with a diagnosis of low-grade chondrosarcoma arising from osteochondroma, considering the clinical and radiological features. After surgery, the shoulder was immobilized with an arm sling for 3 weeks, followed by rehabilitation.

The radiating pain and dysesthesia of his left arm and hand disappeared soon after surgery, and the range of motion of the left shoulder improved to within the normal range. Muscle weakness also recovered approximately 1 month after the surgery. Neither local recurrence nor metastases were observed 3 years later (Figure 4(f)), and there was no functional disability of the left shoulder and arm.

## 3. Discussion

We presented the case of a patient with HME presenting with TOS as a symptom of secondary malignant change in osteochondroma.

The pathophysiology of TOS includes heterogeneous causes that compress neurovascular structures within the retroclavicular space. There are three frequent causes of compression: congenital abnormalities (including bone, fibrous, and muscular anomalies); posttraumatic muscular hypertrophy and scarring; and posture and muscle imbalance [5]. Tumor, as was observed in our case, is rarely the cause of TOS, and in the previously reported instances, the tumor was located on the first rib [1, 2]. Only one report has described a tumor of the clavicle causing TOS [4], although there are a few reports of its causation by the nonunion or malunion of midthird fractures of the clavicle [6, 7]. The pathophysiology for this type of TOS is the depressed and posteriorly elevated proximal tip of the distal fragment, as well as the abundant callus formation at the site, which compresses the brachial plexus and subclavian artery. In our case, the tumor was located at the midshaft of the clavicle and protruded in a posteroinferior direction, compressing the subclavian artery and brachial plexus in a manner similar to that of clavicle fractures. TOS is commonly classified into vascular and neurological forms, and more than 95% of cases are neurological [8]. The angiography in our case revealed that the tumor compressed the subclavian artery, and the brachial plexus was also compressed between the tumor and the thoracic wall, as confirmed during the operation. Therefore, our case included both vascular and neurological forms. TOS symptoms can vary depending on which structures are compressed, and elevating the arm induces the symptoms in most cases. In this case, the atypical symptoms of the left arm and hand were relieved by elevating the arm. Given the variability of the causes related to TOS, many provocative maneuvers have been described to aid in its diagnosis, but the tests have high rates of false-positive and false-negative results [9]. Atypical symptoms were observed in our case, but the symptoms similar to TOS suggested that the cause of the symptoms might exist in the retroclavicular space.

Malignant transformation of osteochondroma in patients with HME is not frequent; the incidence has been reported as about 2% to 5% [3, 10]. The predilection sites of secondary peripheral chondrosarcoma are the skeleton of the pelvis, scapula, and proximal limbs, and the frequency of malignant transformation of osteochondroma in the clavicle was about 1% [11]. Considering the low incidence of malignant transformation, the periodic examination of each osteochondroma in patients with multiple osteochondromatosis is not always needed. Most malignant transformations of osteochondroma are detected by worsening pain, functional disability, and/or growing mass, specifically after maturation of the skeleton. In our case, the tumor was very large when the patient presented at our hospital, and because the retroclavicular space is difficult to access by palpation, we have to consider periodic radiographic examination in order to detect the malignant transformation of osteochondroma earlier if it develops in this area. In addition, we have to pay close attention to the neurological symptoms because these symptoms depend on the tumor locations, which are mostly in enclosed spaces where nerves pass through, such as the spinal canal, retroclavicular space, or carpal tunnel.

In summary, this is the first case of TOS associated with secondary chondrosarcoma of the clavicle, and the neurological symptoms, such as TOS, may possibly be the key symptoms for diagnosing this type of malignant transformation.
